# Raptorial appendages of the Cambrian apex predator *Anomalocaris canadensis* are built for soft prey and speed

**DOI:** 10.1098/rspb.2023.0638

**Published:** 2023-07-12

**Authors:** Russell D. C. Bicknell, Michel Schmidt, Imran A. Rahman, Gregory D. Edgecombe, Susana Gutarra, Allison C. Daley, Roland R. Melzer, Stephen Wroe, John R. Paterson

**Affiliations:** ^1^ Palaeoscience Research Centre, School of Environmental and Rural Science, University of New England, Armidale 2351, Australia; ^2^ Division of Paleontology, American Museum of Natural History, New York, NY 10027, USA; ^3^ Bavarian State Collection of Zoology, Bavarian Natural History Collections, Munich, Germany; ^4^ Yunnan Key Laboratory for Palaeobiology, Institute of Palaeontology, Yunnan University, North Cuihu Road 2, Kunming 650091, People's Republic of China; ^5^ The Natural History Museum, Cromwell Road, London SW7 5BD, UK; ^6^ Oxford University Museum of Natural History, Oxford OX1 3PW, UK; ^7^ Institute of Earth Sciences, University of Lausanne, Lausanne CH-1015, Switzerland; ^8^ Faculty of Biology, Biocenter, Ludwig-Maximilians-Universität München, Planegg-Martinsried, Germany; ^9^ GeoBio-Center, Ludwig-Maximilians-Universität München, Munich, Germany; ^10^ Function, Evolution and Anatomy Research Lab, School of Environmental and Rural Science, University of New England, Armidale, New South Wales 2351, Australia

**Keywords:** Cambrian, *Anomalocaris*, predation, kinematics, biomechanics, computational fluid dynamics

## Abstract

The stem-group euarthropod *Anomalocaris canadensis* is one of the largest Cambrian animals and is often considered the quintessential apex predator of its time. This radiodont is commonly interpreted as a demersal hunter, responsible for inflicting injuries seen in benthic trilobites. However, controversy surrounds the ability of *A. canadensis* to use its spinose frontal appendages to masticate or even manipulate biomineralized prey. Here, we apply a new integrative computational approach, combining three-dimensional digital modelling, kinematics, finite-element analysis (FEA) and computational fluid dynamics (CFD) to rigorously analyse an *A. canadensis* feeding appendage and test its morphofunctional limits. These models corroborate a raptorial function, but expose inconsistencies with a capacity for durophagy. In particular, FEA results show that certain parts of the appendage would have experienced high degrees of plastic deformation, especially at the endites, the points of impact with prey. The CFD results demonstrate that outstretched appendages produced low drag and hence represented the optimal orientation for speed, permitting acceleration bursts to capture prey. These data, when combined with evidence regarding the functional morphology of its oral cone, eyes, body flaps and tail fan, suggest that *A. canadensis* was an agile nektonic predator that fed on soft-bodied animals swimming in a well-lit water column above the benthos. The lifestyle of *A. canadensis* and that of other radiodonts, including plausible durophages, suggests that niche partitioning across this clade influenced the dynamics of Cambrian food webs, impacting on a diverse array of organisms at different sizes, tiers and trophic levels.

## 1. Introduction

The Cambrian explosion is epitomized by the development of the first complex marine animal ecosystems and trophic differentiation [1–3], including a surge in predation activity [4,5]. Exemplary among Cambrian predators are the radiodonts—stem-group euarthropods bearing a pair of large, arthrodized frontal appendages and a ventral oral cone with tooth-like serrations—that are often the largest animals within their respective ecosystems [6–16]. Many radiodonts possess frontal appendages that are considered to have been raptorial and able to capture, manipulate and perhaps even masticate prey, before passing food items to the mouth for further processing [6,17–22]. In this context, select radiodonts are hypothesized to have been capable of shell-crushing (durophagous) predation [21–23], but there is still uncertainty as to whether breaking biomineralized prey was achievable using the frontal appendages, paired gnathobase-like structures affiliated with segments transitional between the head and trunk, the oral cone, or a combination of these, depending on the taxon [5,6,24–26].

**Figure 1.**
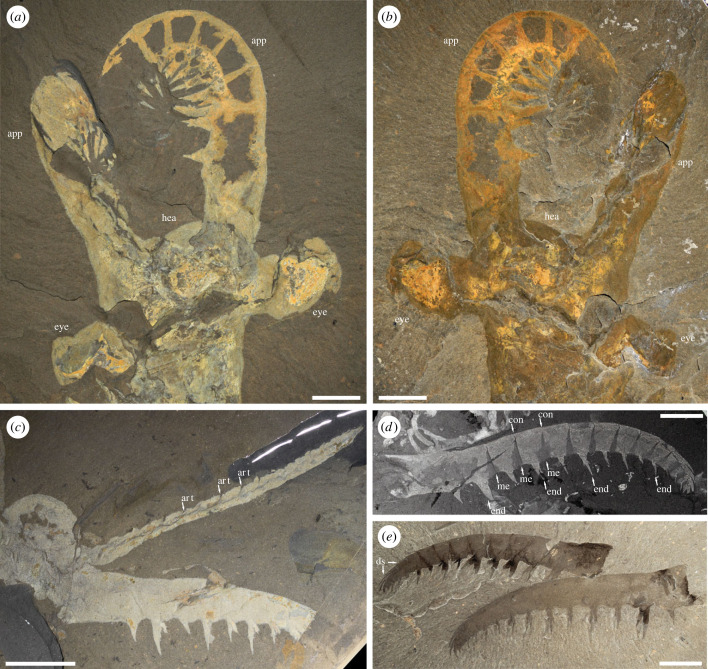
Key examples of *Anomalocaris canadensis* specimens from the Cambrian (Miaolingian, Wuliuan) Burgess Shale of Canada that informed the frontal appendage reconstruction. (*a*,*b*) Close-up of the head of a complete specimen, showing the maximum frontal appendage flexure. (*a*) ROMIP 51212b (counterpart). (*b*) ROMIP 51212a (part). (*c*) Pair of frontal appendages, with one preserved in dorsal view (arrowed) showing the dorsal expression of podomere articulations and indicating a plausible width of the appendage. ROMIP 61650. (*d*,*e*) Large, exceptionally preserved frontal appendages. (*d*) Specimen showing details of condyles, endites and arthrodial membrane; this specimen was used to scale the finite element and computational fluid dynamic models. ROMIP 61675. (*e*) Two appendages that show paired endites on each podomere and distal dorsal spines. ROMIP 62543. app, appendages; art, podomere articulation; con, condyle; ds, dorsal spine; end, endite; eye, lateral compound eye; hea, head; me, membrane. Scale bars: (*a*,*b*) 10 mm; (*c*,*e*) 20 mm; (*d*) 15 mm. (*a*,*c*) Imaged under water. (*b*,*d*,*e*) Imaged under cross-polarized light. (*d*) Image converted to greyscale.

The most iconic radiodont, *Anomalocaris canadensis* from the Cambrian (Miaolingian, Wuliuan) Burgess Shale of Canada (figure 1), has long been suspected as a possible durophage, especially preying upon trilobites [27–32]. Some studies [6,26] have questioned the ability of the *Anomalocaris* oral cone to crush biomineralized prey, whereas another explicitly suggested that both the oral cone and frontal appendages were involved in the flexing and eventual breakage of trilobite exoskeletons [22]. Although recent three-dimensional (3D) modelling has shown that *A. canadensis* frontal appendages had a high degree of flexibility to possibly perform such a task [18], the hypothesis that radiodont appendages functioned the same way as modern raptorial euarthropod appendages [20] and were capable of inflicting damage to biomineralized prey remains to be tested quantitatively.

Here, we provide an accurate 3D digital reconstruction of an *Anomalocaris canadensis* frontal appendage based on exceptionally preserved specimens from the Burgess Shale [9] (figure 1), and subject it to kinematic, biomechanical modelling using finite-element analysis (FEA) and computational fluid dynamics (CFD). These quantitative analyses reveal the morphofunctional capabilities and hydrodynamic performance of the frontal appendages of this iconic Cambrian apex predator, with important implications for Cambrian ecosystems [6,7,9].

## 2. Methods

### (a) Modern analogues for kinematic models

Based on similarities in form and inferred function, Liu *et al.* [20] suggested that the multi-segmented raptorial appendages of whip scorpions (Uropygi) and whip spiders (Amblypygi) [20,33–36] would be useful for understanding radiodont appendages. As such, we developed kinematic models of *Mastigoproctus giganteus* (Uropygi) and *Heterophrynus elaphus* (Amblypygi) as modern analogues. We used one *M. giganteus* specimen housed in the New England Natural History Arthropod collection (NENH-AR), University of New England, Armidale, Australia, and one *H. elaphus* specimen housed in the Zoologische Staatssammlung Arthropod Collection (ZSMA) Munich, Germany, to generate arachnid kinematic models. Both specimens were scanned using micro-computed tomography (micro-CT) at optimized conditions at their respective institutions (see electronic supplementary material, data S1 for more details). Scans were imported into Mimics v. 23.0 (Materialise, Leuven, Belgium). Podomeres of the raptorial appendages were separated with the ‘Segmenting' tool, and muscles and tendons were removed using the same tool. Segmentation was conducted manually, using density difference and morphology of structures in the scan to identify separate podomeres and soft tissue. Separated podomeres were exported as .STL files from Mimics and imported into Geomagic Studio (www.3dsystems.com) for smoothing. The smoothed .STL files were exported as .OBJ files for kinematic analyses in Maya 2020 (Autodesk, San Rafael).

### (b) *Anomalocaris* appendage reconstructions

A 3D reconstruction of the *Anomalocaris canadensis* raptorial appendage was rendered in Zbrush (Pixologic Inc.). The reconstruction was informed by direct examination of fossils, including specimens preserved at various orientations in the rock matrix and with different degrees of flexure, as well as previous appendage reconstructions [6,9,37] (figure 1). To determine the relative proportions of the podomeres, ratios were taken from the most informative fossils (electronic supplementary material, data S2), following a similar approach to De Vivo *et al.* [18]. Appendage ‘inflation' was informed by examining specimens preserved at oblique angles (e.g. Daley & Edgecombe [9], fig. 4.1; figure 1*c*). This approach was needed as the fossils are two-dimensionally preserved and the density difference between fossil and rock matrix in Burgess Shale specimens gives insufficient contrast for micro-CT scans. Although Chengjiang radiodonts (Cambrian Series 2, Stage 3) have been successfully micro-CT scanned [38], the entire appendage cannot be reproduced. As such, the appendage would still need to be reconstructed, and likely retro-deformed [39–41]. We did not reconstruct the arthrodial membrane between podomeres as this membrane would have contracted and extended during appendage motion, and these details cannot be accurately modelled with the available computational capabilities. The reconstruction was exported as an .STL file from Zbrush for import into Geomagic Studio, where it was converted into an .OBJ file. This file was then exported for import into Maya 2020.

### (c) Kinematic models

The models of *Anomalocaris* and the modern analogues were imported as .OBJ files into Autodesk Maya for kinematic analyses [42,43]. Artificial rotation joints were assigned to podomere articulations with the ‘X_ROMM' add-on [43–45]. Bicondylar pivot and hinge joints were constructed for the arachnids based on previous studies [46–48]. While a pivot joint consists of two articulation points across the podomere, a hinge joint consists of two articulation points (condyles) in the same region, either side of the articulation. Furthermore, this is a common joint type in euarthropods [49,50]. For *Anomalocaris*, joints were constructed as bicondylar hinge joints because this morphology is consistent with the V-shaped regions of arthrodial membrane extended between *Anomalocaris canadensis* podomeres (figure 1*d,e*). Joint axes were modelled as long cylinders, the most basic mathematical joints. After building joints, the *srjoints* tool was used to deflect podomeres in conjunction with adjacent segments (following Schmidt *et al.* [43]). Maximum flexure was determined in two ways:

(1) When all proximal and distal podomere margins were in contact with each other—a conservative model of rotation.
(2) When podomeres were allowed to telescope—podomeres could rotate under each other, following De Vivo *et al.* [18]. In this realistic model, maximum flexure was limited by endite morphology.

Collision detection was done by visual assessment. Although automatic clash detection can be conducted using a Boolean-approach [51,52], we used a manual approach here as this method is more accurate and precise for our analyses.

**Figure 2.**
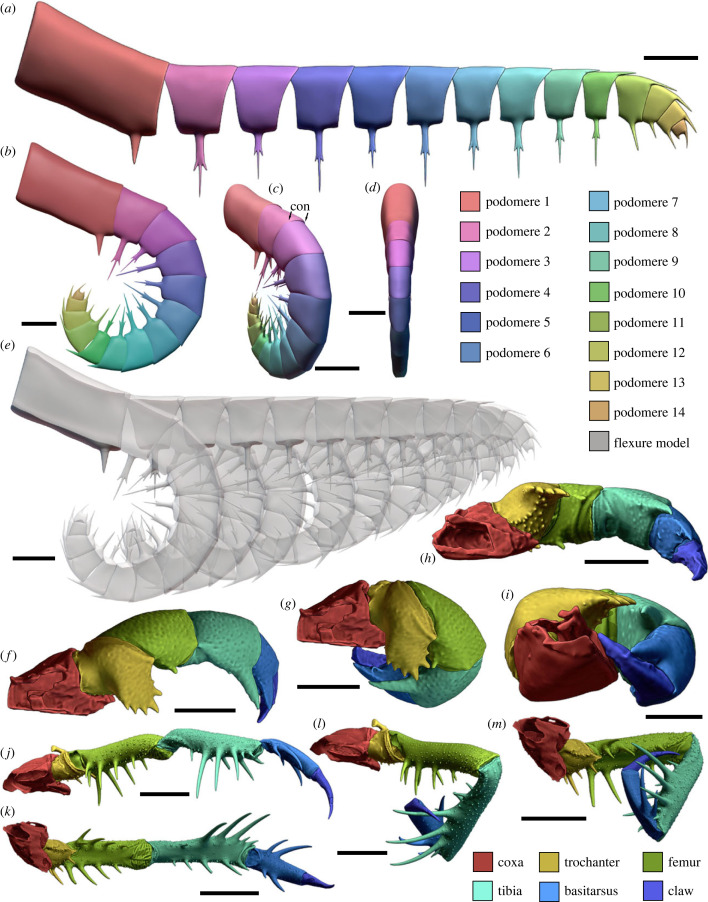
3D kinematic models of the *Anomalocaris canadensis* appendage in comparison with *Mastigoproctus giganteus* (whip scorpion) and *Heterophrynus elaphus* (whip spider) raptorial appendages. (*a*–*e*) *Anomalocaris canadensis* kinematic appendage models. (*a*) Model completely outstretched, colour coded for podomere number. Lateral view. Electronic supplementary material, figure S1. (*b*–*d*) Raptorial appendage maximally flexed. Electronic supplementary material, figure S2. (*b*) Lateral view. (*c*) Oblique orientation. (*d*) Anterior view. (*e*) Illustration of sequential appendage motion from outstretched to maximally flexed. (*f*–*i*) Models of *M. giganteus*. NENH-AR00011. (*f*,*h*) Pedipalp outstretched. Electronic supplementary material, figure S3. (*f*) Dorsal view. (*h*) Medial view. (*g*,*i*) Pedipalp maximally flexed. Electronic supplementary material, figure S4. (*g*) Dorsal view. (*i*) Medial view. (*j*–*m*) Models of *H. elaphus*. ZSMA 20120286. (*j*,*k*): Pedipalp outstretched. Electronic supplementary material, figure S5. (*j*) Dorsal view. (*k*) Medial view. (*l*,*m*) Pedipalp maximally flexed. Electronic supplementary material, figure S6. (*l*) Dorsal view. (*m*) Medial view. Scale bars (*a*,*b*,*d*–*m*) 5 mm; (*c*) 10 mm. con, condyle. Reconstruction of *Anomalocaris canadensis* credited to Katrina Kenny.

Podomeres 11–14 were considered one functional unit as there is no clear fossil evidence (e.g. presence of arthrodial membrane) to indicate they could move independently of each other (figure 2). The maximum extension and flexure states of both the modern analogues and *Anomalocaris canadensis* kinematic models were exported as .STL files to generate 3D PDFs using Tetra4D (Adobe Systems) (electronic supplementary material, data S3–S5 and figures S1–S7). Further, the *A. canadensis* kinematic models were used to inform the finite element and computational fluid dynamic models.

### (d) Finite-element analysis

**Figure 3.**
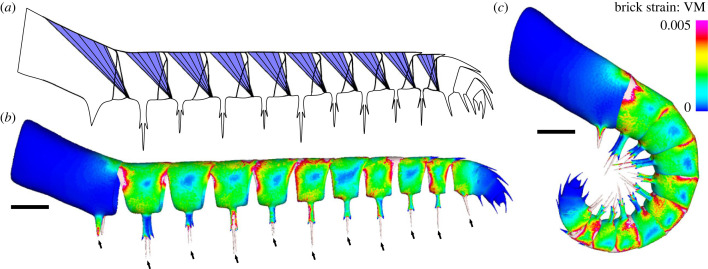
Theoretical biomechanical model and solved *Anomalocaris canadensis* FEMs showing von Mises (VM) brick stress maps in lateral view. (*a*) Proposed *A. canadensis* model showing modelled muscle groups. (*b*) *Anomalocaris canadensis* model outstretched. (*c*) *Anomalocaris canadensis* completely flexed. Scale bars: all 10 mm. Arrows indicate constrained endites.

Details of radiodont frontal appendage musculature, including for *Anomalocaris canadensis*, are unknown, with the only described internal structures being diffuse wide bands of dark material running dorsally along the length of the appendage and narrow dark bands extending into the base of the ventral endites [9, fig. 13]. These were interpreted as possible cavities or chambers within the appendage and are not thought to be musculature, owing to the diffuse preservation that does not resemble the three-dimensionality of musculature seen elsewhere on the *A. canadensis* body [9]. These appendages have homonomous podomeres that adduct in a rotational motion, as shown in the kinematic models (figure 2) [18]. This contrasts with the arachnid appendages used for the kinematic comparisons, as the raptorial appendages of arachnids have fewer and more varied podomeres with a complex combination of muscle groups [53]. As such, muscles in the arachnid analogues are inappropriate for biomechanically modelling the *A. canadensis* frontal appendages. A more comparable analogue for musculature is the oviger appendage of male pycnogonids [54], which has homonomous segments and singular, serially arranged flexor muscles [54]. Hence, for the biomechanical models, we proposed that adduction of *A. canadensis* podomeres used muscle groups that are analogous to muscles within podomeres of pycnogonid ovigers [54]. We also modelled biologically realistic origin and insertion points of these muscles using the origin and insertion points observed in pycnogonid ovigers to model rotation of *A. canadensis* podomeres (figure 3*a*). It is important to note that this is a simplified model, as levator and depressor muscles may also have been present in the *A. canadensis* appendages. However, as we lack any muscle information, we have opted to present a model that requires the fewest assumptions.

For FEA, the kinematic models of *Anomalocaris canadensis* showing the flexed and outstretched positions were imported into Geomagic Studio and scaled to the size of ROMIP 61675 (figure 1*d*), one of the larger frontal appendages of *A. canadensis*. This scaling was performed to determine the strain that one of the larger individuals would have experienced. These models were then exported as .STL files for import into Materialise 3-matic v. 12. Following the kinematic analyses, podomeres 1–10 were considered independent of each other, and podomeres 11–14 were considered one functional unit, as justified above. These podomeres were then solid-meshed as independent homogeneous structures in tet-4 elements in 3-matic and exported as a Nastran file for import into Strand7 (www.strand7.com) FEA software, where the podomere material properties were assigned. We used a Young's modulus of 867 Nmm^–2^ (for sclerotized cuticle: Dalingwater [55]) and a Poisson's ratio of 0.3 (for isometric cuticle; Van der Meijden *et al.* [56]). Proposed muscle origins (figure 3*a*) were tessellated as a group of beam elements onto the Nastran models within Strand7. Origins were considered on the internal surface of the dorsal section of podomeres. Muscle forces were loaded onto three beams directed toward the insertion points. These points were considered the proximal-most ventral section of the subsequent podomere (figure 3*a*) and the beams were used to model muscle bands. Insertions are treated as single, static points at the beam terminus. As the muscle forces are unknown, each beam was loaded with an arbitrary force value of 1 N [57]. A hinge between each podomere and the subsequent podomere was constructed along the dorsal section of the models using two links, with one link on each podomere where there was overlap between podomeres. This imitates a simplified closure of the sections. Finally, each endite between podomeres 1 and 11 was constrained in all directions at the most apical point. This emulated a point of contact with prey. A colour-coded von Mises (VM) microstrain map was generated after solving the model. As in most comparative FEA studies of fossil taxa, the results from the simulations are relative and are therefore not indicative of absolute microstrain values [58]. The loaded Strand7 models for the outstretched and flexed models are presented in electronic supplementary material, data S6 and available from https://osf.io/pqc4r/.

### (e) Computational fluid dynamics

The flexed, intermediate and outstretched models, as proposed from the kinematic analyses of the *Anomalocaris canadensis* frontal appendage, were imported into the open-source 3D computer graphics software Blender v. 2.79 (www.blender.org) [59]. These models were used as references for generating simplified appendage models (excluding endites, which were added later) through box modelling in Blender [60]. In each case, a cylinder was taken as the base object, with vertices and edges of the cylinder fitted to the outline of the reference model in different orientations through translation, rotation and scaling. Additional elements were added through extrusion, and similarly translated, rotated or scaled, until the entire appendage had been modelled in this way. Visual inspection confirmed that the simplified Blender models very closely resembled the original detailed appendage models. Models were exported from Blender as .STL files and then imported into Geomagic Studio, where they were converted into NURBS surfaces (.IGS files) using the AutoSurface function.

The simplified appendage models were imported into the simulation software COMSOL Multiphysics v. 6 (www.comsol.com). Endites, consisting of eccentric cones, were added to these models using the geometry tools in COMSOL. Each appendage model (electronic supplementary material, data S7) was then duplicated to give a pair (with a spacing between the models of 15 mm, based on figs 1–3 and 5 in [9], and fig. 5 in [61], scaled to the size of large appendages), and placed in a computational domain. The domain consisted of a cylinder measuring 240 mm in diameter and 1800 mm in height, which extended at least 3× the length of the models upstream, 10× the length of the models downstream and 5× the size of the models in all other directions. A Boolean operation was used to subtract the models from the domain, and the material properties of liquid water (density = 1000 kg m^–3^, dynamic fluid viscosity = 0.001 kg m^−1^ s^−1^) were assigned to the space surrounding the models. An inlet with a normal inflow velocity was specified at one end of the domain and an outlet with a static pressure equal to zero was defined at the opposing end. No-slip boundaries were assigned to the model walls, with slip boundaries used for the sides of the domain. Previous work suggests that *Anomalocaris canadensis* predominantly fed on prey in open waters [18], and so we assumed the animal was swimming well above the seafloor (and thus not influenced by the ground effect) in all our analyses. The domain was meshed using free tetrahedral elements, with a refinement area used to create a finer mesh in parts of the domain close to the models. A sensitivity analysis was carried out using the outstretched appendage models to determine the coarsest mesh at which the results (i.e. drag forces) were independent of the mesh size (i.e. less than 1% difference from the finest mesh, see electronic supplementary material, data S8), and this was then selected for use in all subsequent simulations. The Reynolds-averaged Navier–Stokes equations were solved using the two-equation shear-stress transport turbulence model [62], with a stationary solver used to obtain a steady-state solution. We simulated inlet velocities of 0.4, 0.7 and 0.9 ms^–1^ for each pair of models. These values were selected based on the swimming speeds calculated by Usami [63] for *Anomalocaris* individuals with lobe widths of 60–70 mm, as was the case for large *A. canadensis* specimens with comparably sized appendages to those modelled herein [9].

CFD results were visualized as plots of flow velocity magnitude and pressure distributions across the appendage models. In addition, we computed the drag forces (*F_D_*) and lift forces (*F_L_*) exerted by the fluid on the appendages and then calculated the drag coefficients (*C_D_*) and lift coefficients (*C_L_*) using the following formulae:

$$C_{D}=\frac{2F_{D}}{\rho U^{2}A}$$

and

$$C_{L}=\frac{2F_{L}}{\rho U^{2}A}$$

where ρ is the fluid density (kg m^–3^), *U* is the inlet velocity (ms^–1^) and *A* is the surface area (m^2^). CFD results files are available from https://osf.io/pqc4r/.

This study was restricted to analysing the hydrodynamics of the frontal appendages, for which adequate fossil data and modern morphofunctional analogues are available to constrain reconstructions [6,9,37]. Attempting to produce an accurate 3D model of the entire body would introduce many assumptions, as very little is known about the three-dimensionality of the *Anomalocaris canadensis* trunk and associated body flaps, despite the availability of several complete body specimens [9].

## 3. Results

Rotation in *Anomalocaris canadensis* appendage kinematic models (figure 2*a–e*; electronic supplementary material, figure S8) reflects a similar degree of motion to that observed in modern raptorial arachnid analogues (figure 2*f*–*m*). The *A. canadensis* models show that by constraining podomere rotation at the dorsal condyles, the most distal podomeres initiate rotation (figure 2*e*; electronic supplementary material, figure S8). Models either prohibiting or allowing telescoping between adjacent podomeres demonstrate that appendage closure resulting in no space between endite tips was not possible (figure 2*b*,*e*; electronic supplementary material, figure S8).

FEA using serially arranged muscles (figure 3*a*) show that both flexed and outstretched (unflexed) *Anomalocaris canadensis* models experienced higher VM microstrain at constrained endites (figure 3*b*,*c*; electronic supplementary material, data S6). These regions of higher VM microstrain extend along the constrained endites up to the auxiliary spines and along the proximal sides of podomeres 2–11 (i.e. regions proximal to points of rotation). Podomeres 1 and 12–14 have lower VM microstrain, reflecting the limited rotation modelled for these podomeres. The presence of similar VM microstrain distributions in both flexed and outstretched models suggests that during attacks, these regions of each podomere would have experienced overall higher degrees of plastic deformation and higher microstrain, especially at the points of impact with prey.

**Figure 4.**
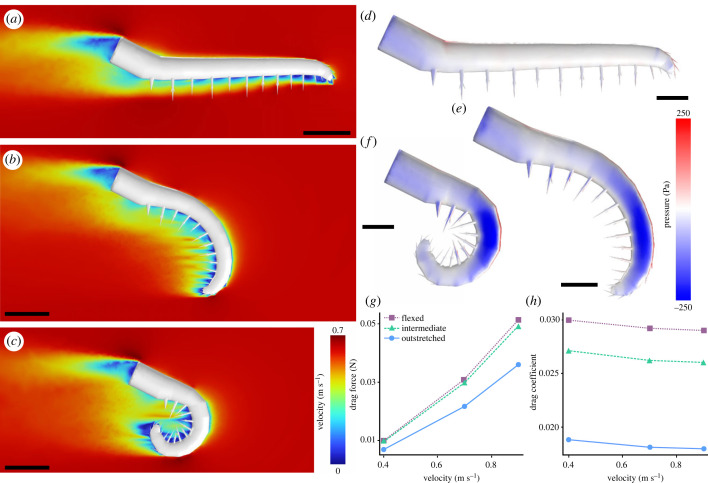
Computational fluid dynamics simulation results for the *Anomalocaris canadensis* frontal appendages. (*a*–*c*) Two-dimensional surface plots of velocity magnitude for the (*a*) outstretched, (*b*) intermediate and (*c*) flexed models. (*d*–*f*) Surface pressure plots for the (*d*) outstretched, (*e*) intermediate and (*f*) flexed models. The inlet velocity was 0.7 ms^–1^ and the ambient direction of flow was from right to left. Lateral view of left appendage. (*g*) Drag forces for the outstretched, intermediate and flexed models (left appendages only) at inlet velocities of 0.4, 0.7 and 0.9 ms^–1^. (*h*) Drag coefficients for the outstretched, intermediate and flexed models (left appendages only) at inlet velocities of 0.4, 0.7 and 0.9 ms^–1^. Scale bars: (*a*–*c*) 20 mm; (*d*–*f*) 10 mm.

CFD simulations showed that the results were almost identical for both models in each pair (electronic supplementary material, data S9), and so we only describe the results for the left appendage model below. In all the models—consistent with theoretical expectations—a sharp velocity gradient was developed in the immediate vicinity of the appendage (the boundary layer), with a region of low-velocity flow (the wake) downstream of the model (figure 4*a*–*c*; electronic supplementary material, figure S9). There was a close correspondence between areas of higher pressure across the appendage and lower fluid velocity, especially clear at the leading edge (figure 4*a*–*f*; electronic supplementary material, figures S9 and S10). Also consistent with theoretical expectations, the drag forces generated by the appendage models increased with increasing velocity, whereas the drag coefficients slightly decreased as velocity increased (figure 4*g*,*h*; electronic supplementary material, data S9). The lift forces and coefficients were negative in all cases (electronic supplementary material, figure S11 and data S9). However, some notable differences between the three appendage models were also apparent. In particular, the drag produced by the outstretched model was considerably lower than for the flexed and intermediate models at all simulated inlet velocities (figure 4*g*,*h*; electronic supplementary material, data S9). These differences were most pronounced at high inlet velocities, indicating that the drag reduction resulting from an outstretched position was more substantial at higher swimming speeds.

## 4. Discussion

Kinematic models comparing *Anomalocaris canadensis* to the modern arachnid analogues demonstrate effective raptorial appendage motion in the former, which is consistent with previous modelling [18]. These models also demonstrate that *A. canadensis* was unable to completely enclose the appendage when fully flexed. While this would have imposed limitations on prey size, which would have varied throughout the ontogeny of *A. canadensis* [18], it would have also prevented endites from impacting on each other and potentially causing damage. Despite this limitation, the high degree of dexterity in the frontal appendages suggests that *A. canadensis* could have efficiently grasped prey items of varying sizes and morphologies. However, the higher VM microstrain distribution along the thin, elongate endites predicts that the appendages were not reinforced sufficiently for exerting high levels of force onto prey. As such, the endites would have been damaged if used to attack biomineralized taxa (e.g. trilobites). Indeed, if biomineralized prey were regularly targeted, it is expected that stunted or injured endites would be commonly observed among *A. canadensis* appendage specimens. To date, only one specimen that shows such a putative injury has been documented (see Daley & Edgecombe [9], fig. 12.3). Biomechanical and fossil evidence therefore indicates that *A. canadensis* was very unlikely to have preyed upon calcified trilobites or other biomineralized taxa. This has two major implications for *A. canadensis* predation. Firstly, *A. canadensis* would have been limited to soft (non-biomineralized) prey, which may have been pierced by the endites during attacks [64]. Secondly, the evidence for durophagy (e.g. skeletal damage and shelly coprolites) in Cambrian deposits [5] should not be attributed to *Anomalocaris* or closely allied radiodonts. This is further supported by the morphology of the *A. canadensis* oral cone, which has pliable, weakly sclerotized plates that could not fully occlude [9,26]. Rather than radiodonts, recent studies instead identify gnathobase-bearing artiopodan euarthropods as predominant durophages in Cambrian ecosystems [39,40].

Construction of the FEMs presented here required key assumptions that must be addressed. These include cuticle properties, muscle reconstructions and muscle force. Importantly, these assumptions are presented within the framework of modern arthropod analogues. The proposed material properties (Young's modulus and Poisson's ratio) replicate values used in previous FEAs of Cambrian arthropods from the Burgess Shale [39,40], the latter informed by exoskeletal cuticle of horseshoe crabs [55]. Preservation of *Anomalocaris canadensis* and other radiodont frontal appendages is consistent with cuticular sclerotization comparable to co-occurring non-biomineralized euarthropods. Muscles are based on sea spider oviger appendage musculature, as there are few modern arthropod appendages that possess homonomous podomeres [54]. Finally, the force value of 1 N per muscle beam was used, resulting in each muscle exerting a 3 N input force, comparable to muscle forces exerted by other arthropods [39,65]. These last two assumptions were needed as muscles must be constructed to run the FEA and muscles are not preserved in frontal appendage fossils. These assumptions mean that absolute numerical values of strain cannot be interpreted in any meaningful way. We have instead focused on where the strain is concentrated in the FEMs, allowing us to determine where intense mechanical strain was experienced in the appendage. Such applications of FEA on extinct morphologies inherently lack exact modern comparisons, but are useful for understanding stress and strain distributions in modern and extinct animals [66,67]. Importantly, the higher strain along the endites is comparable to FEMs of other fossil arthropods with elongated spines on their feeding appendages [39], highlighting that these morphologies are sub-optimal for grabbing and crushing reinforced prey.

CFD results reveal that the frontal appendages of *Anomalocaris canadensis* had an optimized orientation for rapid swimming in open waters, suggesting that it was an agile predator, consistent with hydrodynamic studies of the body flaps and tail fan [63,68]. The outstretched posture produced reduced drag and thus would have lowered the energetic cost of locomotion [69], potentially enabling the animal to swim more efficiently at higher speeds. Although our analyses do not account for the contribution of the body to the drag produced by the entire animal, previous studies have demonstrated that appendages can represent important components of the total drag coefficients of aquatic animals [70] and hence they would be expected to have had a notable impact on the cost of locomotion for *A. canadensis*. We infer that *A. canadensis* preferentially positioned its appendages in an outstretched position to maximize swimming speed, for example, during acceleration bursts to capture prey, similar to modern predatory water bugs [71].

Our computational analyses, coupled with observations of the anatomy of *Anomalocaris canadensis*, especially regarding the functional morphology of the frontal appendages, body flaps and tail fan [9,18,26,63,68], predict this taxon was an active nektonic apex predator that fed on soft prey living within the water column above the benthos. This is further supported by evidence for acute vision in a closely related species from the Emu Bay Shale of South Australia [61,72]. It is likely that the eyes of *A. canadensis* were also adapted to targeting prey in well-lit waters, which may have excluded it from hunting in darker environments, particularly on the benthos in deep-water settings, but it may have also ventured into shallower waters with illuminated seafloors [73–75]. This, coupled with the possibility that *A. canadensis* would have damaged the ventral endites and dorsal spines of the frontal appendages on the substrate if trying to rapidly grab prey from the seafloor (as indicated by our FEA results), contradicts the idea that it was primarily a demersal predator [30,76], particularly of benthic trilobites [22,27–29,31,32]. Instead, *A. canadensis* had a large diversity of nektonic and pelagic soft-bodied animals to potentially feed upon, including a variety of other euarthropods (especially the common isoxyids and hymenocarines such as *Waptia* and *Canadaspis*), as well as ctenophores, nectocaridids and vetulicolians [73,75,77,78], leaving other Burgess Shale radiodonts (e.g. *Hurdia* [8,79], *Cambroraster* [11], *Stanleycaris* [21] and *Titanokorys* [12]), artiopodans (e.g. *Sidneyia* [80]), and various other predators to exploit the benthos [75].

## 5. Conclusion

Kinematic and biomechanical analyses of *Anomalocaris canadensis* frontal appendages demonstrate that, despite being a raptorial predator, this iconic species was incapable of crushing biomineralized prey with these feeding structures. This evidence, coupled with our analyses of the hydrodynamics of the frontal appendages, suggest that this Cambrian apex predator targeted mobile soft-bodied prey within a well-lit water column. While this excludes *A. canadensis* as a key suspect for attacking benthic trilobites and other hard-shelled prey, radiodonts such as *Amplectobelua* [24], *Ramskoeldia* [25] and possibly *Peytoia* [6,21] may have been better equipped for durophagy. These findings add to a growing body of evidence for niche partitioning among radiodonts [10,12,17,18,20,21,61,81], reaffirming the complexity of Cambrian food webs [4,82], and highlighting the diverse weaponry that had rapidly evolved among early euarthropod predators, which likely drove further anatomical innovation in prey armature [5].

## Data Availability

Virtual 3D PDFs, .STL files, loaded FEA models and CFD simulation files can be downloaded from OSF: https://osf.io/pqc4r/. The data are provided in electronic supplementary material [83].
